# Antibacterial Electrophoretically Loaded Titania Nanotubes on Titanium Alloy Implants Enhance Osseointegration

**DOI:** 10.3390/pathogens14111072

**Published:** 2025-10-22

**Authors:** Julia Fischer, Deborah J. Hall, Meghan M. Moran, Adrienn Markovics, Peter H. Pennekamp, John L. Hamilton, Markus A. Wimmer

**Affiliations:** 1 Tribology Laboratory, Department of Orthopedics, Rush University Medical Center, Chicago, IL 60612, USA; juliaflh@aol.com (J.F.); john_l_hamilton@rush.edu (J.L.H.); 2 Anaesthesiologie, Intensiv- & Notfallmedizin, Sana Krankenhaus Gerresheim, 40625 Duesseldorf, Germany; 3 Implant Pathology Laboratory, Department of Orthopedics, Rush University Medical Center, Chicago, IL 60607, USA; deborah_hall@rush.edu; 4 Department Anatomy & Cell Biology, Rush University Medical Center, Chicago, IL 60607, USA; meghan_moran@rush.edu; 5 Division of Rheumatology, Department of Internal Medicine, Rush University Medical Center, Chicago, IL 60612, USA; adrienn_markovics@rush.edu; 6 Klinik fuer Orthopaedie und Unfallchirurgie, Alterstraumatologie, Cellitinen-Krankenhaus St. Hildegardis, 50931 Koeln, Germany; ppennekamp@sthildegardis.de

**Keywords:** antibacterial coating, osseointegration, titania nanotubes, gentamicin, chitosan, electrophoretic loading, animal test, mouse femur implant, pull-out test

## Abstract

Primary hip and knee arthroplasties are common surgeries in the U.S., with periprosthetic joint infection (PJI) being the leading cause of implant revision. Systemic antibiotics often fail to achieve sufficient local concentrations, driving interest in localized drug delivery. Titanium (Ti) implants modified with titania nanotubes (TNTs) provide an increased surface area for drug loading and controlled release. Previous studies have shown that gentamicin-loaded TNTs inhibit *Staphylococcus aureus* growth in vitro without compromising osteoblast viability. This study investigated the effect of gentamicin–chitosan (GC)-coated TNT implants in a murine model, hypothesizing a positive impact on osseointegration. Titanium alloy (Ti6Al4V) wires were anodized to form TNTs and then coated with gentamicin–chitosan (GC) via electrophoretic deposition. Implants (Bare, TNT, TNT+GC; n = 30) were inserted bilaterally into femoral canals of C57BL/6J mice. After > 1 month, osseointegration was assessed by histological point counting, scanning electron microscopy (SEM)-based areal analysis, and mechanical pull-out testing. ANOVA was used to identify differences between groups, and linear regression was applied to account for harvest time, bone contact area, and anatomical section. Bone area fraction (BAF) around the implant measured by the SEM–areal method was significantly higher around TNT+GC (18.4% ± 1.1) and TNT (16.5% ± 1.4) versus Bare (9.0% ± 2.3) (*p* < 0.0028) implants. The maximum fixation strength was higher for TNT (0.878 ± 0.175 N/mm^2^) and TNT+GC (0.853 ± 0.215N/mm^2^) when compared to bare implants 0.316 ± 0.082 N/mm^2^) (*p* = 0.048 and *p* = 0.050, respectively). No significant differences appeared between TNT and TNT+GC. These findings indicate that GC coatings on TNT implants do not impair osseointegration and may even enhance bone–implant integration. Such coatings may therefore provide dual benefits, offering antibacterial protection while improving bone fixation, making them a promising strategy for PJI prevention. Further long-term studies are needed to confirm durability and clinical translation.

## 1. Introduction

Primary hip and knee arthroplasty ranks among the top five most frequently performed procedures each year across all surgical disciplines in the US [1]. The most common causes of failure requiring revisions after total joint arthroplasties are infection, aseptic loosening, periprosthetic fracture, instability, wear, pain, implant failure, and arthrofibrosis. Among these, periprosthetic joint infection (PJI) is the leading cause for the revision and re-revision of implants [2]. PJI occurs in approximately 1–2% of primary arthroplasties and 3–10% in revision arthroplasty [3,4], making it a major clinical and economic burden. Current PJI prevention and treatment strategies include intravenous and oral antimicrobial therapies. Such systemic procedures are accompanied by the disadvantage that only a small amount of the drug might reach the location of infection [5]. To overcome this limitation, localized drug delivery systems have emerged as a promising alternative.

Titanium (Ti) and its alloys, owing to their biocompatibility and tunable surface chemistry, are ideal candidates for localized drug delivery applications [6]. Ti surfaces form a stable titanium dioxide passive layer (TiO_2_, Titania) that prevents adverse chemical reaction with body fluids [7]. The thickness and morphology of this oxide layer can be precisely tailored through electrochemical anodization [8]. Under defined electrolyte compositions and electrochemical parameters, the titania layer can self-organize into nanoscale tubular structures—titania nanotubes (TNTs)—which have attracted considerable interest as localized drug delivery platforms [9,10,11,12,13,14]. TNTs substantially increase the surface area and enable enhanced drug loading, for instance, via electrophoretic deposition (EPD) technology of agents such as antibiotics, like gentamicin (G) and chitosan (C), for a slower release [15]. Gentamicin was chosen for its well-established broad-spectrum antibiotic properties, which are used in orthopedic applications [16,17]. Chitosan provides an interesting combination of complementary advantages, including minimal foreign body reactions, an intrinsic antibacterial characteristic, non-toxicity, and biodegradability, as well as its flexibility in controlling the release of drug agents [18]. In vitro investigations have demonstrated that EPD-loaded gentamicin–chitosan (GC) coatings on titanium implants with TNTs inhibit *Staphylococcus aureus* (*S. aureus*) proliferation in agar diffusion assays while preserving osteoblast viability [15].

To evaluate whether these antimicrobial effects translate to a living system, a murine model of implant-associated infection was developed using a femoral intramedullary implant [19]. In this model, a titanium implant (Ti; 0.6 mm diameter × 8 mm length) is surgically placed in the femur, and the surgical site is inoculated with *S. aureus*. In untreated mice, infection peaks by day 3 and persists at and around the implant for the duration of the observation period. Notably, implants with TNTs and EPD of GC were able to prevent infection at the implant and surrounding tissue in a murine PJI model [20].

Beyond their antimicrobial properties, TNTs promote osteogenic differentiation in vitro, while, e.g., additional gentamicin loaded by means of a lyophilization method and vacuum drying had no adverse effect [13]. Recent in vivo studies further corroborate these findings: histological analyses and mechanical pull-out testing of specimens demonstrate that TNTs improve bone bonding approximately nine-fold, with the rate of mineralization around TNT surfaces being roughly three times higher than that of untreated titanium surfaces [21].

Based on these findings, we hypothesized that GC-loaded Ti implants would enhance osseointegration. To test this hypothesis, we employed a preclinical mouse model of bilateral femoral implants. Bone–implant contact and mechanical fixation strength were assessed, with outcomes including histological evaluations of bone integration and measurements of pull-out force to determine implant stability. Outcome endpoints were the bone area fraction (BAF = bone area/total area = BA/TA) around the implant for point counting and areal analysis, while the maximum fixation strength was the outcome endpoint for pull-out testing. We did not expect TNTs+EPD-loaded implants to have better or worse outcomes compared to groups with TNTs alone.

## 2. Materials and Methods

### 2.1. Creation of Nanotubes on Titanium Wires

**Figure 1:**
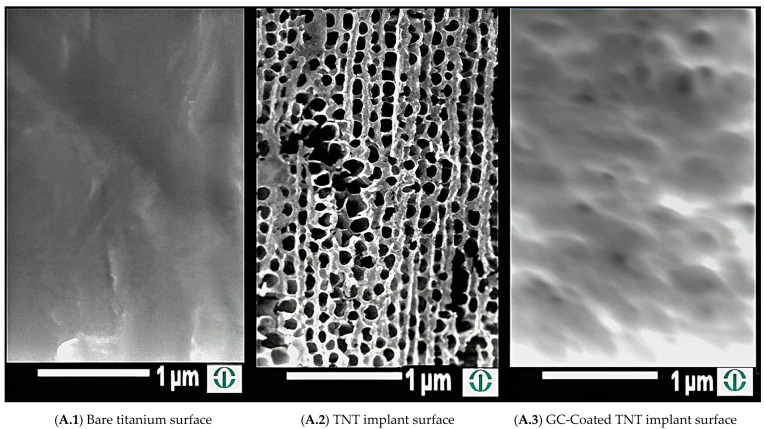
SEM images of implants from the three experimental groups. Note that GC is used throughout the study for the GC coating.

Medical-grade titanium wires (Ti6Al4V, ISO 5832-3 [22]; 0.6 mm diameter, 30 mm length) were obtained from Custom Wire Technologies (Port Washington, WI, USA). Nanotube morphology was created using a two-step electrochemical anodization process in the presence of ammonium fluoride [15]. The first step (60 min anodization) and the second step (30 min anodization) were performed at 70 V. In between steps, the wires were sonicated (Branson 5800, Branson Ultrasonics, Brookfield, CT, USA). This two-step process has been shown to create the most homogenous nanotubes compared to a one-step process, resulting in TNTs approximately 100 nm in diameter and 7 to 12 µm in length, which were observed by means of a scanning electron microscope (JEOL JSM-IT500HR with IT500HR V1.030, Jeol Technics Ltd., Peabody, MA, USA) [15] (Figure 1).

### 2.2. Electrophoretic Disposition on Implants with TNT

EPD is based on the application of a direct current electric field to drive suspended charged particles toward and onto an oppositely charged substrate [23]. This technique was used to coat TNT implants with gentamicin and chitosan.

The TNT-coated wire served as the cathode, and a spiral-shaped platinum wire served as an anode in a two-electrode setup (Interface 1010E potentiostat, Gamry Instruments, Warminster, PA, USA). EPD loading was performed in two consecutive 5 min steps at a constant potential of −5 V. The solution for the first step contained both gentamicin (100 mg/mL) and crosslinked chitosan (2 mg/mL) in ethanol, while the solution for the second step only contained chitosan (2 mg/mL) and ethanol. Based on our light microscopical analysis, this produced an approximately 20 μm thick gentamicin–chitosan (GC) coating on the TNT wire’s surface. For each produced batch, coatings were randomly inspected with SEM to ensure uniform coating deposition across implants.

### 2.3. Implantation and Retrieval Protocol

This study was conducted in accordance with the Declaration of Helsinki and approved by the Rush University Medical Center Institutional Animal Care and Use Committee (IACUC, Rush University Medical Center, Comparative Research Center, 1735 W. Harrison St., Suite 206, Chicago, IL, USA). The following procedures complied with the Guide for the Care and Use of Laboratory Animals (Institute of Laboratory Animal Resources, National Academy of Sciences, Bethesda, MD, USA). In vivo experiments were based on an existing mouse model with femoral implants, as published earlier [19,20,24]. In this study, thirty 12-week-old C57BL/6J male mice (Jackson Laboratories, Bar Harbor, ME, USA) were randomly assigned to three groups (n = 10 per group) and received bilateral implants. No infections were included. Group 1 received bare Ti6Al4V wires (‘Bare’), and group 2 received Ti6Al4V wires with TNTs (‘TNT’), while group 3 received Ti6Al4V wires with electrophoretically deposited gentamicin–chitosan-loaded TNTs (TNT+GC).

On the day prior to surgery, both hindlimbs were shaved using electric hair clippers and Nair hair removal cream (Nair, Church & Dwight Co., Ewing, NJ, USA). On the day of surgery, mice were weighed, injected subcutaneously with buprenorphine (0.1 mg/kg) analgesic, and anesthetized with 2% isoflurane.

**Figure 2:**
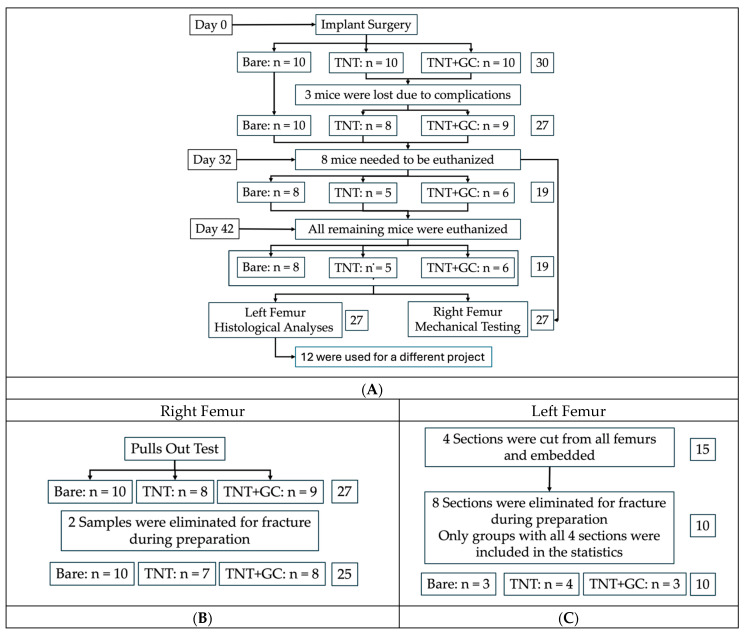
(**A**). Study overview: Mice received bilateral femoral implants on day 0 and were sacrificed on day 32 or 42. X-ray imaging was performed after implantation and before harvest to confirm correct implant positioning. Body weight was monitored throughout the study (before implantation, after 3 weeks, and on harvest day). (**B**). Processing of right femurs. (**C**). Processing of the left femurs. The numbers represent the number of mice used.

**Figure 3:**
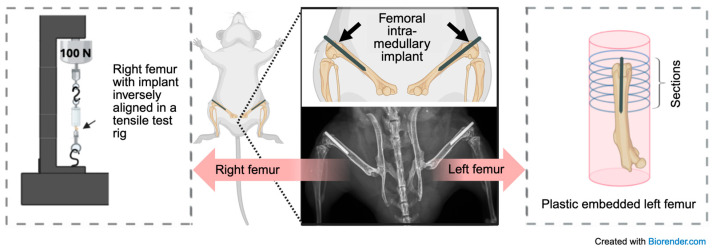
Schematic representation of a mouse with a surgically placed bilateral intramedullary femoral implant and subsequent sample processing. After sacrifice, the right femurs were embedded for mechanical pull-out testing, while the left femurs were processed and sectioned for histological evaluation using the point-count and areal method.

Under aseptic conditions and with the aid of a dissection microscope (Zeiss Stemi 508, Carl Zeiss AG, Oberkochen, Germany), a skin incision was made over the right knee with a sterile Micro Knives scalpel (10315-12, Fine Science Tools, Foster City, CA, USA). A medial parapatellar arthrotomy was performed as previously described [19]. The quadriceps patellar complex was displaced laterally to expose the femoral condyles, and a 25-gauge needle was used to ream the femoral intramedullary canal of approximately 10 mm in length. The (Ti) wire—8 mm long and measuring 0.6 mm (Bare), 0.61 mm (TNT), or 0.64 mm (TNT+GC) in diameter—was placed into the canal retrogradely, leaving 1–2 mm protruding from the distal femur. Implant diameters differed slightly among groups—0.60 mm for Bare, 0.61 mm for TNT, and 0.64 mm for TNT+GC—reflecting additional oxide and coating layers. The GC layer was expected to be gradually released and biodegraded over time as previously reported [15]. The quadriceps muscle was sutured, the patella repositioned, and the incision closed. The same procedure was repeated on the contralateral limb. To ensure proper femoral implant placement, X-ray images were performed postoperatively. Mice were weighed before surgery, at three weeks post-surgery, and at harvest to monitor general health. In accordance with the IACUC-approved protocol and under the supervision of veterinary staff at the Rush Comparative Research Center, animals were continuously monitored for general pain and distress. Since the mice for this work were not infected, it was unlikely that the type of coating affected pain. In total, 8 mice were harvested after 32 days, and 19 mice were harvested 42 days post-surgery, as will be elucidated later. Figure 2 and Figure 3 illustrate the experimental overview and femoral processing steps.

### 2.4. Histological Analyses

#### 2.4.1. Embedding and Sectioning

Left femurs were retrieved on harvest day for undecalcified histology. Specimens were fixed in 10% buffered formaldehyde for at least three days at 4 °C and then placed in 70% alcohol prior to undecalcified plastic embedding. The time of fixation was directly dependent on specimen size and thickness.

The two methyl methacrylate (MMA) solutions (Polysciences, Washington, PA, USA) for infiltration (MMA I) and embedding (MMA II) were prepared as follows: The inhibitor was completely removed from the MMA monomer by washing with 5% sodium hydroxide. The monomer was then washed with distilled water and dried by filtering slowly over anhydrous sodium bicarbonate. Next, the catalyst (0.5% benzol peroxide) was added and dissolved by stirring. The solution of clear MMA monomers with catalysts was then divided into two parts, labeled I and II, and stored in a refrigerator. MMA II was further prepared by completely dissolving 30% of the bead polymer by volume in a solution labeled MMA II.

Dehydration and embedding were carried out over the course of one week in a vacuum. Specimens were dehydrated in a vacuum through a graded alcohol series (70%, 95%, and 100%) and using xylene (each for 24 h). After dehydration, the specimens were infiltrated with MMA I under vacuum and refrigerated for 72 h. For embedding, the MMA II solution was brought to room temperature, and specimens were embedded under vacuum for one hour before being left to polymerize at room temperature.

Polymerized specimens were sectioned transversely into five 1.27 mm slices using a low-speed saw with a diamond-tipped blade (Isomet, Buehler, Lake Bluff, IL, USA). Sections were numbered from distal to proximal. Section 1 was excluded from the analyses due to its proximity to the femoral condyles.

#### 2.4.2. Point-Counting Analysis by Light Microscopy

For point counting, sections 2 and 4 were mounted on slides with epoxy (Buehler, Lake Bluff, IL, USA) and ground to a final thickness of 200 µm. The slides were first dipped twice in 4% hydrochloric acid and then immersed in a toluidine blue/basic fuchsin staining solution (Toluidine Blue O, Pararosaniline Hydrochloride, Fisher Scientific, Waltham, MA, USA). Finally, they were placed in a drying oven at 36 °C for 90 min. After staining, they were thoroughly rinsed in distilled water and dried with highly absorbent paper.

**Figure 4:**
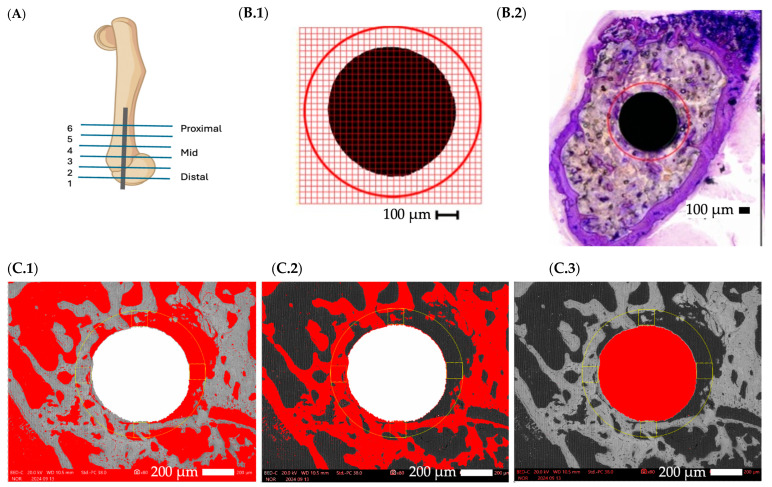
(**A**). Scheme of a left mouse femur displaying obtained sections from distal (1) to proximal (6). Histologic point-counting method scheme (**B.1**) and histological image (**B.2**) showing ROI circle centered over implant and a 25 µm grid overlay. The red circle indicates a 100 µm peri-implant region used for point counting. (**C.1**–**3**). Areal method scheme displaying a yellow ROI circle centered over the implant, indicating a 100 µm peri-implant region used for threshold analysis. Thresholds were set for distinguishing between bone marrow, shown in red in (**C.1**), bone, shown in red in (**C.2**), and implant, shown in red in (**C.3**).

Light microscopy images of the sections were acquired at 200× magnification using Keyence VHX-6000 (Keyence Corp. of America, Itasca, IL, USA). To assess osseointegration, a digital point grid was overlaid on the images in Photoshop (V 25.11.0). The region of interest (ROI) was defined as a circle encompassing the implant and extending 100 μm beyond its perimeter. Points spaced at 25 µm were counted and classified according to whether they intersected bone, bone marrow, or the implant (Figure 4).

#### 2.4.3. Areal Analysis by SEM

To validate the point-counting results, a secondary method was employed. The mid and proximal sections (sections 3 and 4) of the implants were sputtered with carbon and imaged using SEM The obtained images were digitally cleaned from artifacts that could be misinterpreted as bone or bone marrow (Photoshop V25.11.0). As in the point-counting method, the (ROI) was defined as a circular area extending 100 μm beyond the implant’s perimeter (ImageJ 1.54k, Public Domain, NIH, Bethesda, MD, USA). Thresholds for bone, bone marrow, or implant were applied, and the percentage of each category within the ROI was quantified directly (Figure 4).

### 2.5. Mechanical Testing

Pull-out testing of the right femurs was performed using an adapted embedding and testing protocol previously described for a rat model [25]. Immediately after retrieval, all right femurs were frozen at −20 °C. Femurs embedded in cold-cure epoxy (Koldmount; SPI #01352P powder and #01352H hardener, Structure Probe Inc., West Chester, PA, USA) were mixed at a 2:1 ratio. Koldmount was selected because it permits the embedding of moist bone samples without prior drying. The components were mixed for 30 s and then poured into embedding tubes around the femurs, leaving the distal femur and implants exposed. After one hour at room temperature, the specimens were placed in a 4 °C fridge overnight. Pull-out testing was completed by gripping the implant in a chuck mounted to a 100 N load cell in the material testing machine (MTS System, Criterion 43, Eden Prairie, MN, USA). Force–displacement curves were used to determine the maximum force at failure. One pull-out test was performed per right femur. The maximum fixation strength was then calculated by dividing the maximum force at failure by the nominal implant surface area (bone contact area = BCA). The BCA was computed from the implant diameter and the measured length of the implant in bony contact, calculated by subtracting the exposed implant length from the known total implant length. The maximum fixation strength served as a proxy measure of osseointegration.

### 2.6. Statistical Analysis

The assumption of normal distribution was assessed by Shapiro–Wilk and D’Agostino–Pearson tests. Homogeneity of variances was evaluated using the Brown–Forsythe test. Outcome variables were BAF around the implant for histological analyses and for obtaining the maximum fixation strength for mechanical testing.

To identify differences between groups (Bare, TNT, and TNT+GC) in mechanical testing and histological data, one-way ANOVA was performed, followed by Tukey’s multiple comparison test as a post hoc analysis.

Three mice were lost due to skin irritations before implantation, which left 27 mice for analysis. Additionally, not all mice could be harvested at the six-week (day 42) time point and needed to be euthanized at day 32 (n = 8). Early harvest had a comparable impact across all groups (bare = 2; TNT = 3; TNT+GC = 3), ensuring the validity of intergroup comparisons. Nevertheless, harvest time, bone contact area (BCA), and anatomical section were treated as confounders. A linear regression model was fitted in RStudio (Posit, PBC (2024), Version 2024.09.1+394). Model assumptions were verified, and the adjusted R^2^ was reported. An additional linear hypothesis test was conducted to directly compare treatment groups 2 and 3.

ANOVA served as the primary approach to evaluate group differences, while linear regression models provided secondary, confirmatory analyses. Baseline regression models were first estimated without covariates, followed by models adjusted for harvest time and, where applicable, BCA in mechanical testing and section (which implicitly controls for the method) in histological analyses. This stepwise approach was used to evaluate model robustness.

After embedding for histology, 8 sections were eliminated due to specimen damage, leaving 52 remaining sections for analysis. For histological analyses, a balanced dataset was used. To maintain a balanced dataset and minimize confounding due to incomplete samples, only femurs with all four intact sections (two per method) were included. A total of 40 sections were analyzed per method (Bare: n = 6; TNT: n = 8; TNT+GC: n = 6). Mean values from sections obtained from each embedded femur were compared.

The control linear regression model for histological analyses was estimated using clustered standard errors to account for within-cluster correlation. The linear regression model controlled for harvest time and section number (as a proxy for analysis method).

As part of mechanical analyses, 25 pull-out tests were performed (Bare: n = 10; TNT: n = 7; TNT+GC: n = 8). Two specimens were excluded due to femur fractures that occurred during the exothermic reaction of the embedding process.

Weight control analyses were carried out with a linear regression model in R Studio.

Main analyses and graphs were generated using GraphPad Prism Software (version 10.4.2, 29 March 2025; GraphPad Software, San Diego, CA, USA). Linear regression control models were performed using R Studio (version 2024.09.1+394; R Foundation for Statistical Computing, Vienna, Austria). Data are presented as mean ± SEM. A *p* ≤ 0.05 was considered statistically significant.

## 3. Results

### 3.1. Histological Analysis

#### 3.1.1. Increased Bone Share Fraction Around TNT and GC-Coated Implants Measured via Point-Counting Method

**Figure 5:**
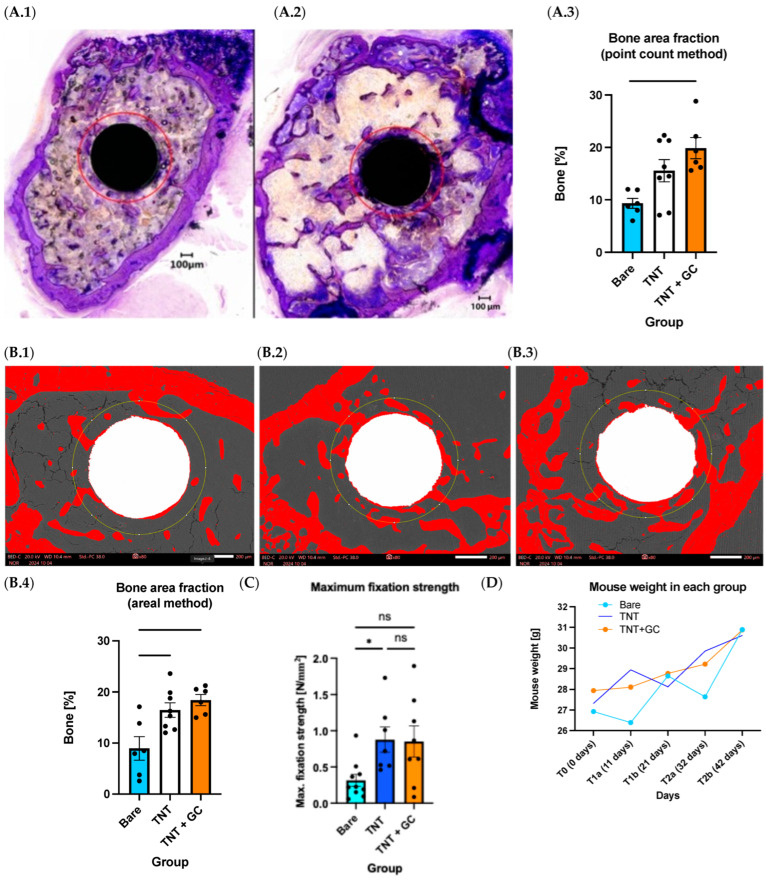
Representative light microscopy images showing BAF in purple around Bare (**A.1**) and TNT+GC (**A.2**) implants. Representative SEM images showing BAF in red around Bare (**B.1**), TNT (**B.2**), and TNT+GC (**B.3**) implants. Note that both light microscopy and SEM images visually show more bone around the TNT and TNT+GC compared to bare implants. These findings are consistent with the quantitative results from the point count (**A.3**) and areal (**B.4**) method, as well as the higher maximum fixation strength of the coated TNT implants: borderline significant (*p* = 0.050) (**C**). (**D**) shows mouse body weight changes in the three groups over time. Data are represented as mean ± SEM; * *p* < 0.05, ** *p* < 0.01.

The highest bone percentage was found around coated implants and TNT implants (Bare (9.3% ± 0.9), TNT (15.6% ± 2.1), and TNT+GC (19.9% ± 2.0)) (Figure 5).

One-way ANOVA revealed a significant effect of the treatment group on bone percentage (F(2,17) = 7.130, *p* = 0.0056, R^2^ = 0.4562). Tukey’s multiple comparisons test showed a significant increase in bone percentage in the TNT+GC group compared to Bare (*p* = 0.0043), while the difference between Bare and TNT did not reach significance (p = 0.0726). No significant difference was observed between TNT and TNT+GC (*p* = 0.2571). The assumptions of normality and homogeneity of variances were satisfied, as all *p*-values exceeded 0.10.

#### 3.1.2. Increased Bone Share Fraction Around TNT and Gentamicin + Chitosan-Coated Implants Assessed via Areal Method

The analysis of the areal method data revealed similar results to the point count data, showing greater bone formation around coated implants and TNT implants compared to the control group (Bare (9.0% ± 2.3), TNT (16.5% ± 1.4), and TNT+GC (18.4% ± 1.1)) (Figure 5). One-way ANOVA revealed a significant effect of the treatment group on bone percentage (F(2,17) = 8.447, *p* = 0.0028, R^2^ = 0.4984). Tukey’s post hoc test showed that both TNT (*p* = 0.0119) and TNT+GC (*p* = 0.0035) groups had significantly higher bone percentages than the Bare group. No significant difference was observed between TNT and TNT+GC (*p* = 0.6786). The residuals satisfied the assumptions of normality and the homogeneity of variance (all *p* > 0.28).

#### 3.1.3. Linear Regression Control Model for Histological Analyses (BAF)

In the linear regression model including harvest time as a covariate, the treatment group remained a significant predictor of BAF. Compared to the control group ‘Bare’, both the TNT+GC group (β = 10.002, *p* < 0.001) and the TNT group (β = 6.884, *p* < 0.001) showed significantly higher BAF. Harvest time itself had no significant effect (β = 0.135, *p* = 0.947), suggesting that variability in the timing of sample collection did not substantially influence the measured values. The section number (see Figure 4A), however, remained a significant factor: Section 3 (β = −3.386, *p* = 0.004), section 4 (β = −5.33, *p* < 0.012), and section 5 (β = −2.31, *p* = 0.233) showed lower BAF values compared to section 2. These results suggest systematic differences based on anatomical locations within the femur.

The model explained a moderate proportion of variance (adjusted R^2^ = 0.501). A linear hypothesis test comparing the TNT and the TNT+GC group indicated a trend toward significance (F(1,33) = 3.173, *p* = 0.084). This finding suggests a possible, though not statistically confirmed, difference between these two groups after adjusting for harvest time and section.

Overall, the main treatment effects, particularly the superior performance of TNT+GC, remained robust after adjusting for harvest time and section number (see Appendix A, Table A1).

### 3.2. Pull-Out Analysis

#### 3.2.1. Implants with TNTs and Implants with TNTs Coated with Gentamicin and Chitosan Resulted in Higher Maximum Fixation Strength

The highest implant fixation strength was measured in coated implants and TNT implants compared to bare titanium implants (Bare (0.316 ± 0.082 N/mm^2^), TNT (0.878 ± 0.175 N/mm^2^), TNT+GC (0.853 ± 0.215N/mm^2^)) (see Figure 5).

One-way ANOVA showed a significant difference among the treatment groups (F(2,22) = 4.447, *p* = 0.0239, R^2^ = 0.288). The post hoc Tukey’s test indicated that the TNT group had significantly higher maximum fixation strengths than Bare, while in the TNT+GC group, the difference was borderline significant (*p* = 0.048 and *p* = 0.050, respectively). No significant difference was observed between the TNT and TNT+GC groups (*p* = 0.994).

#### 3.2.2. Linear Regression Control Model for Pull-Out Analysis (Fixation Strength)

In the multiple linear regression model including harvest time as a covariate, treatment group remained a significant predictor. Both TNT (β = 0.530, *p* = 0.016) and TNT+GC (β = 0.524, *p* = 0.078) had a higher fixation strength compared to bare titanium implants. Harvest time was not a significant predictor (β = −0.153, *p* = 0.575). The bone contact area (BCA) also had no significant influence on group differences (β = −0.032, *p* = 0.957). The model explained 17.2% of the variance (adjusted R^2^ = 0.172). The difference between TNT and TNT+GC remained non-significant (*p* = 0.988) (see Appendix A, Table A2).

### 3.3. Weight Results

Mouse weights were monitored as an indicator of general health. Mice started with an average weight of 27.4 g on surgery day, with no significant differences between the groups at any time point (all ns). As expected, weight increased significantly over time regardless of group assignment (see Figure 5; Appendix A, Table A3).

## 4. Discussion

### 4.1. Interpretation of Results

Building on previous studies demonstrating the antibacterial properties of these implants [20], this study aimed to evaluate the osseointegration potential of antibacterial Ti implants with TNT surfaces coated with an EPD GC layer in a murine model. Specifically, we investigated whether these surface modifications would compromise bone–implant integration or increase the risk of implant loosening.

Our findings demonstrated that both TNT-modified and -coated implants enhanced bone integration compared to unmodified control implants [26]. Although the small sample size may have limited the statistical significance, the data consistently indicate that TNTs with EPD-loaded GC do not impair osseointegration. Implants featuring TNTs and implants with GC-coated TNTs improve bone–implant integration without compromising mechanical stability.

Histological assessments using the point-counting method and high-resolution areal quantification via SEM demonstrated a significantly higher bone fraction in proximity to coated implants. Additionally, pull-out tests revealed an increased maximum fixation strength in the TNT and TNT+GC groups compared to bare implants, indicating improved mechanical stability and osseointegration.

Even TNTs without any antibiotic coating enhanced osseointegration, consistent with previous animal studies [21,27,28,29], which reported improved bone response to nanotopographically modified titanium surfaces. As demonstrated in [13], nanoscale surface topography strongly influences osteoblast adhesion, spreading, and differentiation, thereby promoting early bone formation. This effect may be attributed to the unique nanotube topography and chemical properties of the TNT layer. Previous studies have also shown that anodized Ti surfaces containing phosphorus or calcium phosphate form microporous oxides (0.1–2 µm) that enhanced osteoblast adhesion and proliferation without toxicity while maintaining stable alkaline phosphatase (ALP) activity [30]. Moreover, human fetal osteoblasts cultured on thermally or micro-arc oxidized titanium surfaces exhibited significantly higher ALP expression compared to untreated titanium alloy, indicating that the oxide layer acts as a bioactive stimulus for osteoblast differentiation rather than being inert [31]. Overall, the native titanium oxide layer on titanium alloys actively promotes osteoblast differentiation.

Regarding coated TNT surfaces, the following question arises: What mechanisms explain the enhanced mechanical stability observed in GC-coated TNT implants?

Several studies have identified key processes contributing to this improvement: The additional positive effect observed in the TNT+GC group may be partially explained by the osteoblast-stimulating properties of chitosan described in the literature. Our results align with prior reports of chitosan’s positive impact on mouse osteoblast growth and differentiation, as well as bone gene expression in human mesenchymal stem cells [32,33,34]. Moreover, chitosan has been shown to preferentially support initial osteoblast attachment and the spreading of mouse osteoblasts compared to fibroblasts, further promoting bone formation [35].

The effect of gentamicin on osteogenic cells remains debated. Several in vitro studies report no detrimental effects on osteoblast viability, even at high concentrations or when used in chitosan-based coatings [15,36]. Some studies even suggest improved osteoblast viability and osteoconductivity with gentamicin-loaded nanotubes [37]. However, a concentration-dependent response has also been observed, with high doses inhibiting cell growth, while lower doses promote proliferation and differentiation of human mesenchymal stem cells [38].

The combined antibacterial properties of gentamicin and chitosan likely play a key role in reducing infection-related complications and supporting osseointegration in our murine model. These coatings may prevent bacterial colonization and biofilm formation, thereby limiting inflammation and creating a favorable environment for bone healing [20].

Overall, these findings are in line with our results, which demonstrated improved osseointegration and enhanced the mechanical implant stability of GC-coated TNT implants. While these results are consistent with trends reported in the literature, they should be interpreted cautiously until validation by larger, long-term in vivo studies.

### 4.2. Limitations

There are several limitations in this study. First, histological and SEM-based quantifications were not performed on the same histological sections, preventing definite conclusions on whether the increased bone fraction observed in the most distal section (Figure 4A, femur section 2) reflects physiological differences, such as proximity to the knee joint, or methodological variation. The results indicate that the anatomical location within the femur influences measured bone fractions. While both methods produced consistent findings, the SEM-based areal quantification yielded smaller variances and may provide greater accuracy, as it analyzes the full ROI rather than sample points. However, this assumption remains unproven, since the two methods were not applied to the same sections. Nonetheless, due to its higher efficiency and potentially higher precision, the SEM method may be preferable for future studies.

Second, the statistical power of some tests may have been limited by sample size, as indicated by borderline significant *p*-values (e.g., *p* = 0.05). These trends suggest possible differences that could reach significance in larger cohorts. Additionally, longer follow-up periods may be necessary to detect long-term effects.

Third, in alignment with existing studies in the literature using this mouse model, only male mice were used, which may limit the generalizability of the findings. Future studies should incorporate both sexes to evaluate potential sex-related differences in bone healing and osseointegration.

### 4.3. Conclusion and Future Directions

In this murine model, it was demonstrated that the potential benefits of the antibacterial GC coating are not associated with any compromise in osseointegration. In fact, the use of coated TNT implants resulted in improved implant–bone contact, suggesting that these implants may have a positive effect on osseointegration.

Future investigations should aim to validate these findings in larger animal models and under physiological gait loading conditions. Further research is also needed to elucidate the biological basis for the observed enhancement in osseointegration, particularly the role of chitosan in osteoblast activation. Ultimately, long-term in vivo studies will be required to assess the durability of the antibacterial effect and the long-term mechanical integration of such implants.

The combination of gentamicin with chitosan in implant coatings offers dual benefits, such as effective antibacterial activity and the support of osseointegration of implants, as evidenced by reduced infection rates and trends in better in vivo bone–implant contact [15,20].

In the future, TNT implants loaded with GC have the potential to provide sustained antibiotic delivery while simultaneously enhancing bone–implant integration and reducing biofilm formation, which may significantly improve outcomes for prosthetic implants. Future studies with larger sample sizes and longer follow-up, including the precise quantification of bone–implant contact via techniques such as micro-CT, are required to confirm the long-term efficacy and durability of EPD-loaded TNT implants.

## 5. Patent

A pending patent (PCT/US2023/018967) related to work associated with this study has been filed.

## Data Availability

The data will be available upon request.

## Abbreviations

The following abbreviations are used in this manuscript:

ALP	Alkaline phosphatase;
BAF	Bone area fraction = bone area/total area = BA/TA;
BCA	Bone contact area;
C	Chitosan;
EPD	Electrophoretic deposition;
GC	Gentamicin + chitosan;
G	Gentamicin;
MMA	Methyl methacrylate;
PJI	Periprosthetic joint infection;
ROI	Region of interest;
SEM	Standard error of the mean and scanning electron microscopy;
TNT	Titania nanotube.

## Appendix A

**Table A1:** BAF [39] around implants.

t-Test of Coefficients (Balanced Sample) Including Control for Harvest and Method		
	Baseline Model	Specification Model
(Intercept)	9.143 **** (1.067)	11.855 **** (1.578)
TNT	6.874 **** (1.723)	6.884 **** (1.877)
TNT+GC	10.002 **** (1.360)	10.002 **** (1.385)
Harvest		0.135 (1.999)
Section 3		−3.386 *** (1.110)
Section 4		−5.330 ** (1.992)
Section 5		−2.313 (1.903)
Count	40	
R-squared	0.439	0.501
**F Test: Linear Hypothesis Test: TNT − TNT+GC = 0**		
*F*(1,37) = 3.851, *p* = 0.0573		*F*(1,33) = 3.173, *p* = 0.0841

Data are represented as follows: estimate and significance code, standard error in brackets. Signif. codes: 0.0001 ‘****’; 0.001 ‘***’; 0.01 ‘**’.

**Table A2:** Maximum fixation strength, N/mm^2^.

t-Test of Coefficients (Full Sample) Including Control for Harvest and Bone Contact Area (BCA), mm^2^		
	Baseline Model	Specification Model
(Intercept)	0.316 *** (0.086)	0.483 (0.889)
TNT	0.563 ** (0.208)	0.530 *** (0.201)
TNT+GC	0.538 ** (0.246)	0.524 * (0.283)
Harvest		−0.153 (0.268)
Bone contact area (BCA)		−0.032 (0.575)
Count	25	
R-squared	0.223	0.172
**F Test: Linear Hypothesis Test: TNT+GC − TNT = 0**		
*F*(1/22) = 0.007, *p* = 0.934		*F*(1/20) = 0.0003, *p* = 0.988

Data are represented as follows: estimate and significance code, standard error in brackets. Signif. codes: 0.001 ‘***’; 0.01 ‘**’; 0.05 ‘*’.

**Table A3:** Weight monitoring.

t-Test of Coefficients (Balanced Sample) Including Control for Time Point of Measurement					
		Baseline Model		Specification Model	
(Intercept)		28.451 **** (0.525)		27.069 **** (<0.001)	
TNT		0.242 (0.849)		0.385 (0.901)	
TNT+GC		0.489 (0.489)		0.599 (0.558)	
As factor day 21				0.553 *** (0.398)	
As factor day 32				1.622 **** (0.432)	
As factor day 42				3.453 **** (0.480)	
Count		81			
R-squared		−0.016		0.338	
**F Test: Linear Hypothesis Test: TNT − TNT+GC = 0**					
*F*(1,78) = 0.115, *p* = 0.736			*F*(1,74) = 0.073, *p* = 0.7883		

Data are represented as follows: estimate and significance code, standard error in brackets. Signif. codes: 0.0001 ‘****’; 0.001 ‘***’.
